# Association between particulate air pollution exposure during pregnancy and postpartum maternal psychological functioning

**DOI:** 10.1371/journal.pone.0195267

**Published:** 2018-04-18

**Authors:** Perry E. Sheffield, Rosa Speranza, Yueh-Hsiu Mathilda Chiu, Hsiao-Hsien Leon Hsu, Paul C. Curtin, Stefano Renzetti, Ashley Pajak, Brent Coull, Joel Schwartz, Itai Kloog, Rosalind J. Wright

**Affiliations:** 1 Department of Environmental Medicine and Public Health, Icahn School of Medicine at Mount Sinai, New York, NY, United States of America; 2 Department of Pediatrics, Icahn School of Medicine at Mount Sinai, New York, NY, United States of America; 3 The Institute for Exposomics Research, Icahn School of Medicine at Mount Sinai, New York, NY, United States of America; 4 Oregon Health & Science University School of Medicine, Portland, OR, United States of America; 5 Department of Medical and Surgical Specialties, Radiological Sciences and Public Health, Università degli Studi di Brescia, Brescia, Italy; 6 Department of Environmental Health, Harvard T.H. Chan School of Public Health, Harvard University, Boston, MA, United States of America; 7 Department of Geography and Environmental Development, Faculty of Humanities and Social Sciences, Ben-Gurion University of the Negev, Beer Sheva, Israel; Utah State University, UNITED STATES

## Abstract

Postpartum psychological functioning impacts both women’s health and outcomes in children. Lower income, ethnic minority women may be at particular risk for adverse postpartum mental health outcomes. Studies link ambient air pollution exposure with psychological dysfunction in adults although this association has not been examined among postpartum women.

**Methods:**

We studied associations between prenatal exposure to particulate matter with diameter ≤ 2.5 μm (PM_2.5_) and postpartum psychological functioning in a lower income, ethnically mixed sample of urban US women enrolled in a pregnancy cohort study. Analyses included 557 mothers who delivered at ≥37 weeks gestation. Daily estimates of residential PM_2.5_ over gestation were derived using a satellite-based spatio-temporally resolved model. Outcomes included the Edinburgh Postnatal Depression Scale (EPDS) score from 6 or 12 months postpartum and subscale scores for anhedonia, depressive and anxiety symptoms. Associations were also examined within racial/ethnic groups. Distributed lag models (DLMs) were implemented to identify windows of vulnerability during pregnancy.

**Results:**

Most mothers had less than a high school education (64%) and were primarily Hispanic (55%) and Black (29%). In the overall sample, a DLM adjusted for age, race, education, prenatal smoking, and season of delivery, we found significant associations between higher PM_2.5_ exposure in the second trimester and increased anhedonia subscale scores postpartum. In race stratified analyses, mid-pregnancy PM_2.5_ exposure was significantly associated with increased total EPDS scores as well as higher anhedonia and depressive symptom subscale scores among Black women.

**Conclusions:**

Increased PM_2.5_ exposure in mid-pregnancy was associated with increased depressive and anhedonia symptoms, particularly in Black women.

## Introduction

Current estimates suggest that 10–20% of U.S. postpartum women experience depressive or anxiety disorders, impacting both women’s health and outcomes in their children.[1,2] Identifying modifiable risk factors for adverse postpartum psychological functioning is thus an important public health focus.

Notably, a diagnosis of postpartum depression includes the presence of either depressed mood reflecting high negative affect or anhedonia reflecting low positive affect.[3,4,5,6] Evidence increasingly demonstrates that while there is some overlap, the underlying neurobiological mechanisms and risk factors may differ between these specific psychiatric symptom profiles.[1] Moreover, certain populations are disproportionately burdened. For example, epidemiological data demonstrate that prevalence rates of anxiety, depressed mood, and anhedonia, are elevated in urban ethnic communities, particularly among Black and Hispanic mothers,[5,7,8] and among women of lower socioeconomic status.[9] Other data suggest that the subjective experience of affective states varies by culture and race/ethnicity.[10] Taken together, these lines of research underscore the need to distinguish specific symptom profiles in order to more comprehensively assess risk, particularly among racially/ethnically diverse postpartum women.

In animals, sub-chronic and longer-term exposure to ambient pollution causes depressive and anxiety-like symptoms in adult mice. [11,12,13] In human non-pregnant adult samples, increased exposure to ambient particulate matter, nitrogen dioxide, and ozone have been associated with adverse neuropsychological functioning, including anxiety and depressive symptoms[14,15,16] and dispensing of psychiatric medication.[17] Studies to date have mostly considered older adults and patients with chronic illness who may be particularly vulnerable.[18] Exposure to ambient pollution in pregnancy can enhance systemic oxidative stress[19] and impact endocrine and immune system disruption[20] which in turn has implications for neuropsychological functioning in women during the perinatal period.[21] No prior study has examined associations between prenatal ambient air pollution exposure and psychological functioning in postpartum women.

Furthermore, the dynamic physiology during pregnancy (e.g., changes in cortisol, other hormones, and neurotransmitters) [22,23] could affect susceptibility to toxic exposures during specific vulnerable windows over the prenatal period. Notably, our group has previously demonstrated that using exposure estimates of ambient air pollution averaged over pregnancy (or by trimester) to examine effects of air pollution on health outcomes reduces the power to detect effects or could result in missed effects altogether.[24]

To begin to address this important research gap, we leveraged an ethnically diverse, lower income urban cohort of pregnant women to examine the association between ambient particulate matter with an aerodynamic diameter ≤2.5 μm (PM_2.5_) and postpartum psychological functioning. Our approach examines ambient air pollution exposure estimates by each week of pregnancy in order to use data driven methods to identify potential sensitive windows. Doing so allows us to explore potential sensitive windows of exposure since the effects of environmental toxins may vary over the course of the pregnancy. Given recent data demonstrating racial/ethnic differences in the prevalence of specific psychological symptoms (e.g., depressed mood, anhedonia, anxiety),[5] we also examined associations in race stratified analyses.

## Materials and methods

These analyses included women enrolled in the Asthma Coalition on Community, Environment, and Social Stress (ACCESS) project, a prospective pregnancy cohort originally established to examine the effects of perinatal stress and other environmental factors on urban childhood asthma risk.[25] Briefly, N = 598 Spanish or English-speaking women receiving prenatal care at two academic medical centers and their affiliated community health centers were recruited from 2002 to 2007. Of these, 557 mothers delivered at ≥ 37 weeks gestation and had data on air pollution exposure and postpartum psychological functioning. Based on screening data, mothers who declined vs. enrolled were slightly less likely to be ethnic minorities (78.2% vs. 80.9% Hispanic or African American, respectively) or to have a high school education or less (57.2% vs. 60.8%, respectively) and slightly more likely to report an income level <20,000 annually (37.1% vs. 35.2%, respectively); there were no significant differences between groups on these covariates. The protocol was approved by the human studies committees at Brigham & Women’s Hospital and Boston Medical Center and written informed consent was obtained in the subjects’ primary language.

### Daily prenatal PM_2.5_ exposure over gestation

Women’s daily exposure to PM_2.5_ over gestation was estimated based on their residence during pregnancy. This calculation was done using a validated satellite-based spatio-temporal model that predicts daily ambient concentrations of PM_2.5_, as detailed previously.[26] In brief, the model uses a mixed model approach by regressing daily surface PM_2.5_ measurements, taken from the U.S. Environmental Protection Agency Air Quality System (AQS) and Interagency Monitoring of Protected Visual Environments Network, with daily aerosol optical depth (AOD) measurements, land-use terms (elevation, distance to major roads, percent open space, point emissions, and area emissions), and meteorological variables (temperature, wind speed, visibility). This PM_2.5_ model was trained with monitoring data collected by AQS and then validated with 10-fold cross-validation and used to predict daily PM_2.5_ at 1 km x 1 km resolution allowing for the estimations of PM_2.5_ to be calculated for each woman’s residential address during her pregnancy.[23] The mean cross-validated R^2^ for the New England sub-region that includes the greater Boston area included in this study was 0.88. To reduce noise created by the day-to-day PM_2.5_ variation, women’s gestational exposure estimates were calculated by averaging daily predictions over each week of the pregnancy.

### Postpartum psychological functioning

Women completed the 10-item Edinburgh Postnatal Depression Scale (EPDS) [27] at 6 and 12 months postpartum in a face-to-face interview. Women were asked about symptoms in the past 7 days including: 1: “able to laugh", 2: “looking forward", 3: “self-blaming", 4: “worrying", 5: “scared", 6: “things get on top of me” (overwhelmed), 7: “difficulty sleeping", 8: “feeling sad or miserable", 9: “crying", and 10: “thought of self-harming". Participants rated each item on a Likert scale ranging from never to very often (scored from 0 indicating the most favorable condition to 3 indicating the least favorable condition for each item).

While originally developed as a uni-dimensional scale for depression, a number of studies indicate that the EPDS captures multiple domains [28,29] with possible variation by race/ethnic subgroups.[30,31] As previously reported, the results from an exploratory factor analysis (EFA) and confirmatory factor analysis (CFA) indicated that a three-factor model including depressive, anxiety, and anhedonia symptom subscales was the most optimal fit in our sample as a whole and across racial/ethnic groups with minimal differences.[32] Item 10 “thought of self-harming" was omitted from the subscale analysis given rare frequency of endorsement (<1%). The anhedonia subscale includes items 1 and 2; the anxiety subscale includes items 3, 4, and 5; and the depression subscale includes items 7, 8, and 9. Item 6 (overwhelmed) grouped with anxiety for the Hispanic subgroup but with depressive symptoms for the Black/white/other subgroups, and was used accordingly in our stratified analyses. Values reported on each of these items are summed to determine subscale scores. EPDS scores among women with available data at 6 and 12 months (n = 288) were significantly correlated (*rho* = 0.44, p = <0.0001). The higher score from assessments at either 6 or 12 months postpartum was used in analyses. Analyses considered the total EPDS score and depressive, anxiety, and anhedonia symptom subscale scores.

### Covariates

Potential confounders previously identified as being related to maternal mental health and air pollution exposure were considered. Women's age, education level (up to high school education or more), and self-reported race/ethnicity (Black, Hispanic, white, other) were ascertained at enrollment. Additionally, we included variables that may co-vary with ambient air pollution exposure, such as prenatal smoking and season of delivery, and that have also been associated with maternal mental health.[33,34] Prenatal smoking status was determined by women’s self-report at enrollment and in the third trimester and was classified as positive if a woman reported smoking at either visit.

### Analysis

Because prenatal air pollution exposure is linked to preterm birth [35] and preterm birth is linked to postpartum depression,[36] we restricted analyses to n = 557 mothers who delivered at ≥ 37 weeks to minimize over-adjustment for a pathway variable. The primary independent predictor was PM_2.5_, and the outcomes of separate models included total EPDS score (range 0–30 points) and “anhedonia” (range 0–6 for all subjects), “anxiety” (range 0–9 for Black and white subgroups; range 0–12 for Hispanic subgroup due to an additional item), and “depressive” (range 0–9 for Hispanic subgroup; range 0–12 for Black and white subgroups due to an additional item) symptom subscale scores.

In order to examine the time-varying association between prenatal PM_2.5_ exposure and postpartum mental health, we applied a data-driven statistical method to identify the sensitive prenatal exposure windows related to postpartum psychological outcomes. Specifically, we fit distributed lag models (DLMs)–an analytic method increasingly being used in pregnancy-related studies that can reduce potential bias from highly temporally resolved exposures (e.g., weekly over gestation) [24]- to estimate the time-varying association between weekly estimated PM_2.5_ level during pregnancy and the outcomes, as detailed previously.[37] In brief, this method incorporates data from all time points simultaneously and assumes that the association between the exposure and outcome at a given time point varies smoothly as a function of time. We fit the model Y_i_ = β_0_+∑ [α_j_AP_ij_]+β_1_x_1i_+…+β_p_x_pi_, where Y_i_ is the measured EPDS score, AP_ij_ is the estimated PM_2.5_ level for week j of pregnancy, and x_1i_, …, x_pi_ are covariates for subject i. DLMs constrain the α_j_’s to vary smoothly over time, in our case by using B-splines to characterize the time dependence. The degrees of freedom of the spline were chosen using AIC.[38] A significant sensitive window was identified when the pointwise 95% confidence bands did not contain 0. All models were adjusted for covariates noted previously. Further, to examine race-specific associations, we also conducted DLMs stratified by race/ethnicity. DLMs were implemented using the dlnm package version 2.2.6 in R Studio (Vienna, Austria) and other analyses were performed in SAS (v9.4, SAS Institute Inc., Cary, NC).

## Results

Table 1 shows the distribution of demographic characteristics, average PM_2.5_ exposure over the entire pregnancy, psychological symptom scores, and other covariates for the entire sample as well as by racial/ethnic groups. Most mothers had less than a high school education (64%); their mean age was 25.8 years; they were primarily Hispanic (55%) and Black (29%) and most did not smoke prenatally (86%).

**Table 1:** ACCESS study participant characteristics.

		N (%)			
Characteristics		Total (N = 557)	White (n = 57)	Black (n = 163)	Hispanic (n = 305)
HS Education	>HS	203 (36%)	30 (53%)	79 (49%)	71 (23%)
	< = HS	354 (64%)	27 (47%)	84 (51%)	234 (77%)
Maternal Age (years; median, IQR)		25.8 (22.3, 31.3)	24.7 (21.4, 31.3)	25.8 (21.6, 31.1)	26.0 (22.8, 31.8)
Season of Delivery	Winter	151(27%)	16 (28%)	43 (30%)	84 (27%)
	Spring	126(23%)	11 (19%)	36 (25%)	71 (23%)
	Summer	126(23%)	9 (16%)	31 (19%)	78 (26%)
	Fall	154(28%)	21 (37%)	53 (26%)	72 (24%)
Smoking during pregnancy	No	477 (86%)	32 (56%)	143 (88%)	275 (90%)
	Yes	80 (14%)	25 (44%)	20 (12%)	30 (10%)
Average PM2.5 throughout pregnancy (μg/m3; median, IQR)		16.5 (12.8, 19.8)	14.6 (11.7, 19.0)	16.2 (12.6, 19.5)	17.0 (12.8, 19.9)
Anhedonia subscale ^a^ (median, IQR)		0 (0, 2)	0 (0, 2)	0 (0,2)	1 (0,2)
Anxiety subscale ^b^ (median, IQR)		2 (0, 5)	2 (0,4)	2 (0,4)	2 (0,4)
Depression subscale ^c^ (median, IQR)		1 (0, 4)	2 (1,8)	3 (0, 6)	1 (0,3)
Total EPDS Score (median, IQR)		5 (1, 9)	6 (2, 11)	6 (2,10)	4 (1,8)

^a^ Anhedonia symptom subscale consists of items 1 and 2 (score range 0–6) all racial/ethnic groups.

^b^ Anxiety symptom subscale consists of items 3,4,5 for Blacks and whites (score range 0–9), and items 3,4,5,6 for Hispanics (range 0–12).

^c^ Depressive symptom subscale consists of items 7,8,9 for Hispanic (score range 0–9), and items 6,7,8,9 for Black and white (range 0–12).

### Distributed lag models: Entire sample

**Fig 1:**
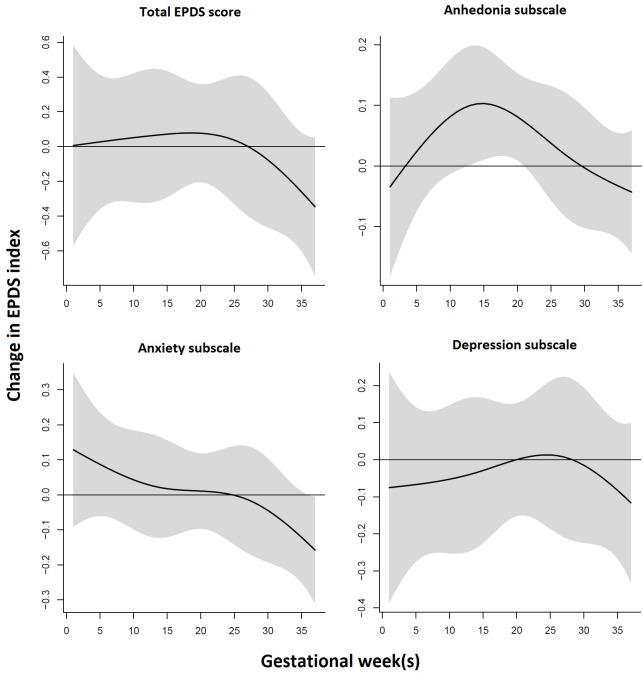
Association between weekly PM_2.5_ exposure and EPDS scores (total score and anhedonia, anxiety and depressive symptom subscales). This figure demonstrates the association between weekly averaged PM_2.5_ during pregnancy and postpartum EPDS total and subscale scores using a distributed lag model assuming week-specific effects, adjusting for race, education, age, prenatal smoking status and season of delivery. The y-axis shows the change in EPDS score in relation to a 10 μg/m3 increase in PM_2.5_ level; the x-axis depicts gestational weeks of the pregnancy. The solid line shows the predicted change, and the gray area indicates the 95% confidence interval. A sensitive window is identified when the estimated pointwise 95% confidence interval does not include 0.

Using AIC, we found the best fit from a distributed lag with 4 degrees of freedom. Fig 1 shows the association between prenatal ambient PM_2.5_ exposure levels and postpartum total EPDS and subscale scores in separate models using DLMs adjusted for race, education, age, prenatal smoking, and season of delivery. We observed a statistically significant sensitive window of PM_2.5_ exposure (estimated for a 10 μg/m^3^ increase in PM_2.5_ exposure) for elevated anhedonia subscale scores during mid-pregnancy, specifically gestational weeks 13 to 20. No statistically significant sensitive window of prenatal PM_2.5_ exposure was found in relationship to the total EPDS score or the other subscale scores.

### Distributed lag models: Race-stratified analysis

**Fig 2:**
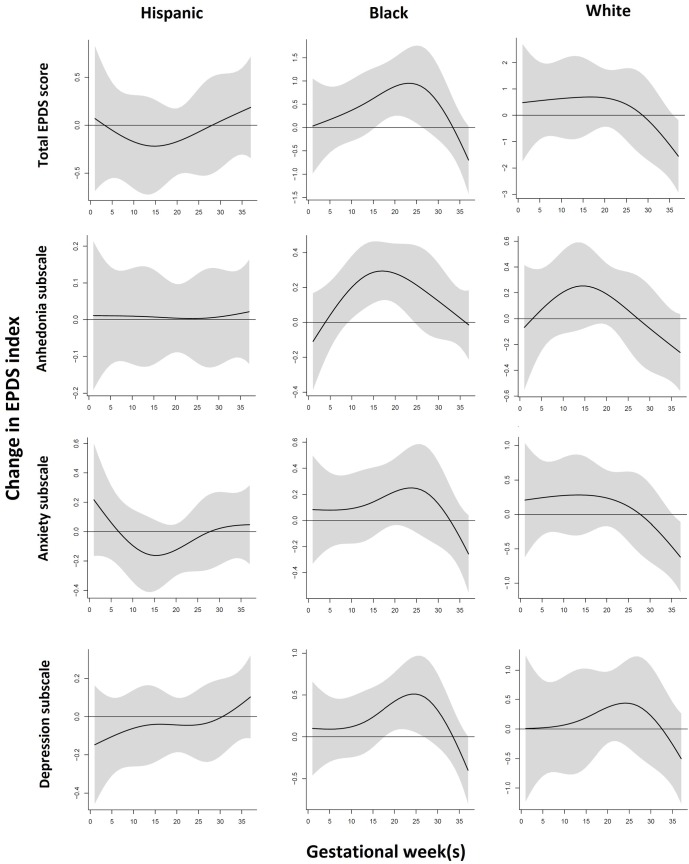
Association between weekly PM_2.5_ exposure and EPDS scores (total score and anhedonia, anxiety and depressive symptom subscales) stratified by race/ethnicity. Within each stratum, this figure demonstrates the association between PM_2.5_ exposure during pregnancy and postpartum EPDS total and subscale scores using a distributed lag model assuming week-specific effects, adjusting for education, age, prenatal smoking status and season of delivery. The y-axis shows the change in EPDS score in relation to a 10 μg/m3 increase in PM_2.5_ level; the x-axis depicts gestational weeks of the pregnancy. The solid line shows the predicted score change, and the gray area indicates the 95% confidence interval. A sensitive window is identified when the estimated pointwise 95% confidence interval does not include 0.

Fig 2 shows the findings from DLMs examining prenatal ambient PM_2.5_ exposure and maternal postpartum EPDS total and subscale scores, stratified by race/ethnicity. In the Black subgroup, we found significant windows of PM_2.5_ exposure (estimated for a 10 μg/m^3^ increase in PM_2.5_ exposure) at 10–24 weeks gestation with anhedonia subscale scores, at 18–26 weeks gestation with depression subscale scores, and at 16–26 weeks gestation with EPDS total scores. The effects in Whites showed a similar, but not significant pattern. There was no significant pattern in Hispanic women; in addition, the shape of the distributed lag plot differed from that of Black and white women.

## Discussion

This is the first study to examine associations between exposure to ambient fine particulate matter during pregnancy and maternal psychological functioning in the first year postpartum. We found associations between mid-pregnancy ambient fine particulate matter exposure and increased postpartum anhedonia symptoms when we considered the sample of ethnically diverse, lower income, urban mothers as a whole. In stratified analyses, effects were most evident in Blacks. Among Black women, increased exposure to PM_2.5_ was significantly associated with higher total postpartum EPDS scores as well as higher scores on the depressive and anhedonia symptom subscales with minor variability in the identified window of vulnerability in pregnancy across these outcome measures.

Notably, we leveraged weekly address-specific PM_2.5_ exposure estimates and DLMs in order to explore this concept of windows of vulnerability for maternal health during the prenatal period. Significant associations were observed primarily in the second trimester. Critical timing of exposure for environmental factors, or windows of vulnerability to exposures during pregnancy in relationship to later maternal health outcomes remains minimally explored and even the theoretical mechanism needs better refinement. A more definitive characterization of vulnerable windows may provide insight into underlying mechanisms when coupled with our understanding of psychopathology in the perinatal period and environmentally induced physiological changes that may underlie observed associations. Mechanisms that may underlie these associations were beyond the scope of the current analysis and will need to be examined in future research.

Additionally, a better understanding of risk and resilience factors is need to further explore the different patterns of associations between air pollution and psychological functioning by racial/ethnic groups. Among the factors to further explore are perinatal factors such as method of delivery, postpartum factors such as breast feeding, as well as larger community and societal factors such as religiosity, partner and larger social support networks, and stress. The pattern of association between PM_2.5_ and EPDS scores observed among Hispanic women differed from the patterns observed among Black and white women. This difference further supports the idea of different phenotypes of psychological functioning among different racial/ethnic groups and also suggests potentially different resilience factors such as social support, community expectations, and other protective factors that warrant further exploration.

Of the three subscales examined, only anhedonia and depressive symptoms were associated with increased exposure to particulate pollution in this study. This association is consistent with the emerging understanding of the role of inflammation in mediating anhedonic behaviors via impacts on neurotransmitter synthesis and accumulation of neurotoxic compounds.[39] However, anxiety has also been linked to inflammatory cytokines and other markers of immune imbalance.[40] In other research, prenatal exposure to particulate air pollution has been associated with decreased gene expression in brain-derived neurotrophic factor (BDNF) [41] and reduced levels of maternal BDNF prenatally has been implicated as a biomarker of postpartum depression.[42] Future research should examine underlying mechanisms beyond inflammation (e.g., oxidative stress, altered gene expression). Moreover, it has been pointed out that symptoms of anhedonia may be commonly overlooked in clinical evaluations in comparison with more overt symptomatology such as depressed mood.[6] Thus, consideration of these different presentations has implications both in research and in the clinical setting if we are to understand the full impact of environmental exposures on postpartum psychological functioning in women. A more nuanced understanding of the specific neuropsychological sequelae of air pollution remains an area of in need of increased investigation.

There are a number of notable strengths of the current analysis. These include the reasonably large sample of lower income ethnically mixed inner-city mothers, the high spatial resolution of the particulate matter exposure estimates coupled with the advanced statistical modeling that allowed us to use data driven methods to identify windows of vulnerability, and our broader assessment of psychological functioning using the EPDS subscales. We also acknowledge some limitations. The exposure estimate was unable to account for the actual amount of time a woman spent at her residence and while ambient air quality is a strong driver of indoor air quality, indoor PM_2.5_ concentrations may have differed resulting in exposure misclassification. Given the growing evidence that indoor air quality is often worse than outdoor air quality, our ambient air exposure estimates are likely conservative. We hypothesize that any misclassification is non-differential and would thus bias our results toward the null. However, indoor air quality is an important and potentially worsening exposure given the changing climate and should be the focus of further study to develop appropriate interventions.[43] While we adjusted for a number of known confounders of the association between ambient particulate matter and maternal mental health, there may be unmeasured confounders we did not consider. For example, we were unable to adjust for pollutants other than PM_2.5_ or other concomitant chemical exposures in these data, specifically ones that may be correlated with PM_2.5_ exposure and on a causal pathway for maternal psychological functioning during the postpartum period. Additionally, while limiting to term births, we were not able to control for other birth outcomes or infant-specific health issues which could potentially be on the causal pathway between *in-utero* air pollutant exposures and maternal psychological functioning. Specific experiences that may differ based on race/ethnicity, for example racism, may moderate the effects of chemical pollutants on mental health outcomes which also may explain differences across racial groups.[44] Future research in sociodemographically diverse samples should consider these additional factors with larger sample sizes to facilitate more formal examination of air pollution mixtures (multiple pollutants) as well as modifying factors. These findings may not be generalizable to populations with differing sociodemographic makeup nor directly comparable to studies that consider average air pollution exposures over the entire or trimesters of the pregnancy. However, our DLM analytic approach reduces the risk of potential bias or missed effects altogether when exposure windows are arbitrarily assigned. [24]

In summary, this study objectively elucidated sensitive prenatal windows and examined racial/ethnic differences for the association between PM_2.5_ during pregnancy and maternal postpartum psychological functioning. Our findings suggest that exposure to particulate air pollution during specific windows of pregnancy may be associated with higher risk of negative postpartum mental health outcomes in women–particularly anhedonia and depressive symptoms. Black women may be at higher risk. While this study adds to the growing literature linking ambient air pollution with mental health outcomes in adults, more studies are needed to replicate these findings in other populations, particularly with respect to potential windows of vulnerability for the mother during pregnancy, and to further elucidate underlying mechanisms.

## Supporting information

S1 Data File ACCESS cohort data. This excel workbook provides a minimal dataset including all variables analyzed in this analysis. A data dictionary is provided on worksheet two of the excel workbook.
(XLS)
